# Practical Psychology1Address to the Alumni Association of the University of Buffalo, February 22, 1900.

**Published:** 1900-04

**Authors:** Frederick Peterson

**Affiliations:** (Class of ’79), of New York, President of the New York Neurological Society; president of the board of managers of the Craig Colony for Epileptics; chief of clinic, department for nervous and mental diseases, Columbia University


### BUFFALO MEDICAL JOURNAL.

VOL. XXXIX.—LV. APRIL, 1900. No. 9.

### Original Communications.

### PRACTICAL PSYCHOLOGY.[1]

BY FREDERICK PETERSON, M. D. (Class of '79), of New York,
President of the New York Neurological Society; president of the board of managers of the Craig Colony for Epileptics; chief of clinic, department for nervous and mental diseases, Columbia University.

YOUR committee which has honored me, and made me grateful by this kind invitation to address you, made the condition that I talk to you about practical psychology. At first I demurred to this, for the subject suggested a little story which Matthew Arnold related in a lecture of his delivered in Buffalo some seventeen years ago. An English school boy being requested to give a practical explanation of the line, "Canst thou administer to a mind diseased," wrote "Can you give medicine to a lunatic." It seemed to me that this sort of practical psychology, therapeutics for the insane, would be a melancholy subject for me to discuss at a gathering of this kind, and I should have rejected it altogether had not the thought subsequently occurred to me that practical psychology after all includes far more than this, indeed almost the whole region of human culture.

Practical psychology is the dominant feature of modern education. It has to do with all the problems of sociology. It is the ruling spirit of the politician. There is no limit to its expansion, its imperialism, in the entire domain of human knowledge and intercourse. Therefore I shall take as my text only that small part of practical psychology with which the physician as to do—that which relates to the interpretation of facts in his science, and which is so deeply interwoven with the phenomena brought before his eye, such as clinical and pathological experience, the tricks of spiritualism, mind-reading and legerde-

1. Address to the Alumni Association of the University of Buffalo, February 22, 1900.

main, the manifestations of hypnotism, dreams and telepathy, and the fatuities of christian science, faith-cure, theosophy, osteopathy and other forms of quackery. Are these things real or are they not? Do we believe them or do we not? Be they actual and credible, upon what do we found our judgment? What is the evidence?

All of these questions must be decided from the standpoint of the value of Human Evidence, and I shall take as my text in this brief address this paramount subject of Human Evidence. It would take too much time and more of the critical faculty than is at my disposition to properly set this subject before you, but at least I may briefly outline what to me are the main conditions modifying the value of evidence and vitiating our inferences and judgments. With things that are known and accepted by all mankind we have none of this difficulty. The accumulated experience of ages has thoroughly sifted the evidence, so that we know the earth to be round, the moon to be a satellite, mathematics to be an exact science, death to be our human lot. But as to the things that are unknown, we stand in the shadows upon shifting sands. We listen to the sanguine or fraudulent assertions of others, our senses are full of illusions, our emotions lead us astray, our longings and preconceptions seize any propitious circumstance with which to prop and stay our wandering minds.

Now what are some of the sources of error in the critical balancing of testimony presented to us? There are two obviously important factors in an inquiry of this nature; first, the quality of the phenomenon observed, and secondly the character and quality of the observer. The things with which we have to deal belong to the domain of the unknown. They are things concerning some of which we are able to grasp certain dim outlines, certain foreshadowings, certain penumbræ, so to say, of the truth, but concerning many of which we can have only surmise, hypotheses, theories, superstitions or unsupported beliefs. They form the "million acres of our ignorance." The observer himself is hampered by the defective and illusory evidence of his more or less imperfect senses, by undisciplined intellect, by the perversions of his hazy memories, by the limitations of his knowledge and experience, by the modifying influences of his emotions, and less often perhaps by a tendency to deliberate deception and misrepresentation of the matters under consideration. We are constantly confronted in our study and practice of medicine with the mass of our ignorance of the things yet to be known and with the defects and limitations of the students of these things. Despite this, however, we are constantly wresting from nature her marvelous secrets and surprising and uplifting the world with our discoveries. It is interesting to examine the practical psychology involved in the elucidation and acceptance of any new fact or problem.

Take any example. Take for instance the matter of cerebral localisation. Compare the knowledge of cerebral localisation in a Hippocrates and in a Horsley, and comtemplate the shifting mass of ignorance concerning this subject during those twenty-three centuries. Think of the thousand preposterous assertions concerning the brain, the thousand absurdities current, the thousand errors promulgated, the work of the multitude of quacks, philosophers, psychologists, physicians, anatomists, physiologists and pathologists, during all those centuries before what seem to us now such simple truths won the acceptance of the modern world! And with regard to the things yet to be discovered in this great unseen and unknown universe about us, the same process of shifting the good from the bad evidence goes ever on in the self same way. The quacks and empirics are with us still, making all sorts of fraudulent claims and ridiculous assertions in relation to phrenology, christian science, faith-cure, osteopathy, and quack medicines of all kinds. We have them in our own profession, adventurers, seekers after notoriety and fortune, exploiting some panacea or other, fake cures for consumption and inebriety, pelvic operations for the cure of psychoses, eye muscle-cutting for the relief of epilepsy, nose-boring for any and every disorder, and so on through the whole category of human diseases. On a plane but little higher than this we have a class of pseudo-scientists, men who occupy a reputable position in the profession and seek by every means to enhance their reputations, even by deliberate falsifications of their observations, proclaiming new discoveries in pathology and therapeutics which they know to be untrue, but which they feel may pass scrutiny for a long time, because of the obscurity of our scientific data and the intricacies of the problems they pretend to solve. Then we come to the body of real workers in every field of medical science, men filled with that eager enthusiasm and burning love of truth which leads them to immolate themselves upon the best of sacrificial altars, that of human progress. It is chiefly upon the errors of these that I wish to dwell, to point out the conditions which often vitiate their evidence.

In the first place there are our imperfect senses; those narrow and dim avenues through which we gain all our knowledge of the outer world. How often have we followed fatuous fires through those shadowy vistas that led not anywhere, how often been misled by illusions that promised so much and redeemed so little. The facts of yesterday become the artefacts of today. The seeming conquerors of the present are overthrown tomorrow. There is always that process of sifting, sifting evidence going on through years and years. Once it was common belief that the neurones were joined together, as are our electric batteries and wires. The work of many laboratories had led to that belief. Suddenly a solitary student announces that the neurones are independent organs, having no direct continuity, functioning only through process contiguity. Again we have the corroborative work of numerous good men in the same field. Then a new arbitrator passes judgment on the labors of his predecessors and reasserts the old idea of continuity, and is in turn supported by the valuable testimony of still others. And now, at this present moment, through the sifting process that has gone on, we are in the position to assume that perhaps both presentations are correct, that there are some neurones that are independent and some which are not. But what a vast amount of work this conclusion represents, what repetitions of experiment and observation, what faults in the eye, what errors of judgment, what warping influences of personal idiosyncrasy!

It is not alone the illusions to which our defective senses are so subject that lead to mistakes and misinterpretations, but there are even greater sources of error in the psychical processes connected with these faulty senses. There are few observers who possess that disciplined intellect, that even temperament, that calm judgment, so necessary to the critical and unprejudiced examination of phenomena. Many medical men have limited horizons, owing to a lack of thorough training and to a want of familiarity with the ascertained data of the many cognate sciences with which their own lines of research are more or less correlated. They are, in other words, deficient in their store of experiences. Another defect lies in want of the imaginative faculty. In studying the unknown we may be handicapped by an inability to conceive of the qualities and character of the phenomena that are hidden, by an unconsciousness of the very existence of such phenomena. It is almost proverbial that most discoveries are stumbled upon, after having stared us in the face for ages. They were always before our eyes, but we had not the imagination to conceive of them. What for instance could be more astonishing to most of us than to be able to talk a thousand miles or more with a friend, or to scrutinise a man's bones with our eyes, not only through his flesh but through his clothes and through a door? While most discoveries are stumbled upon, others are made by scientists who have the conceptive faculty to such a degree that their findings are the result of their brilliant mental prescience and clairvoyance.

Still another fallacy in our interpretation of phenomena is due to the vitiating influence of the emotions upon the judgment. A man's character has been said to be the sum of his ethical emotions, and his judgment of natural phenomena, and especially of what we may term preternatural phenomena, is almost sure to be biased in some degree by the feelings of fear, pleasure, reverence, awe, sympathy or antipathy which they inspire, and these feelings of his are a reflex of the sum of his emotional sensations experienced throughout his whole life in relation to events, customs, religious views and all sorts of convictions and beliefs. Another and very intangible source of error in evidence, particularly in regard to occult phenomena, has been described as the "instinctive tendency of the imagination to dramatic unity and completeness." With less felicity it has also been termed the "mania for finishing." A pathologist begins a piece of difficult work and in his eagerness and impatience to bring it to a successful issue, he unconsciously perverts the evidence presented to fit some preconceived theory or idea, shaping it into a harmonious unity.

There is one profession—that of the reporter—in which a premium is set upon the cultivation of this particular instinct. In that branch of newspaper work, he is most successful who can convert his observations of common place incidents and events into the best "story." In other words, the natural unconscious sophistication of facts by the human mind to make a dramatic unity, is developed in the reporter to the highest degree of conscious artifice. He is no longer, at least in our large cities, a reporter, but a writer of stories, and his work should be judged from the standpoint of art. A comparison of the "stories" in various newspapers founded upon observation of the same incidents, but described by different artists, is instructive, especially to those who are interested in practical psychology; and I mention this here because of the importance of recognising the existence in all of us of this instinct, and because we have an example in this single calling of high tribute paid to the evolution of a cerebral center which in most other callings were better in a state of latency or atrophy. The man who relates a dream, or some extraordinary psychical experience, generally errs in the same way; he unconsciously sophisticates the record from that "instinctive tendency of the imagination to dramatic unity and completeness." And it has actually been demonstrated by the Society for Psychical Research that almost as fixed as law is the tendency, with the lapse of time and the gathering mists of memory, to a greater and greater dramatic exaggeration of marvelous psychic experience through which the narrator has passed.

So to sum up, the chief sources of error in evidence as to even tangible and palpable phenomena and facts, such as those of physiology, cytology, diagnosis, expert testimony, pathology, therapeutics, statistics and the like, are very great and far-reaching. They consist of (1) deliberate fraud, as in all species of quackery; (2) wilful perversion of facts by pseudo-scientists; (3) objective errors through limitation and defect of the senses; (4) limited horizon through defective experience and education; (5) insufficience of the imaginative faculty; (6) vitiation of evidence under influence of the emotions; (7) the innate tendency of the mind to completeness, to dramatic unity in unraveling a mystery.

Here are seven kinds of fallacy with which we are confronted in dealings with material objective things in our own science of medicine, and I have purposely given these details to draw a contrast in the matter of evidence between highly objective natural phenomena and the phenomena which we know as supernatural, intangible, and immaterial. How vastly greater must be the resources of error in our investigation of these most elusive and mysterious subjects! What a wide and tempting field for fraud and imposture! What a fruitful Cagliostrian garden for cultivation by the pseudo-scientists. In this marvelous domain, under coercion by fraud, illusion and hallucination, by intellectual poverty, by feeble judgment and defective memory, by stress and duress of the emotions, by hereditary and preconceived beliefs and superstitions and by that instinctive trend to dramatic finish, the tactile nerve-end, tongue, nose, eye and ear, so inefficient in the material world, may be made to touch the intangible, taste and smell the impalpable, see the imperceptible and hear the inaudible.

You will observe at this meeting tonight the strange deception of the senses which can be practised by the expert in legerdemain. We enjoy these illusions of the senses and we recognise them as such, but in the uninitiated they might well inspire emotions of awe or terror. In an unintelligent age, not so very long ago, people were hanged and burned for performances less mysterious. Many of us possibly, if we encountered these illusions in our every-day life, instead of witnessing them upon the stage, might readily develop morbid superstitions concerning them. As it is they are terrorless, because the prestidigitateurs make a point of advertising their illusions as tricks done for our entertainment.

The mind-reader really does no more, though sometimes it is claimed for him that he is able to actually read your mind. The mind-reader generally differs from the expert in legerdemain only in his pretentions. The one claims ability to read the mind which of course he is unable to do, and the other is an honest deceiver, asking only admiration for the cleverness and artistic merits of his jugglery. Mind-reading, so-called, is a combination of guess work, muscle-reading and jugglery, and these matters are so trite that I need not dwell upon them here.

Hypnotism is a curious phenomenon not yet fully explicable to any of us, though there is less mystery in it than is popularly supposed by the laity. The subject is gradually going through the process of sifting of evidence hereinbefore described. The popular idea that the hypnotic eye is possessed by only a few chosen individuals, that crimes may be committed by the subject under the influence of another, that almost any disease may be cured by this measure, that subjects may be hypnotised at a distance by telepathic influence, and so on, are simply fallacies, given currency by the sophisticated "stories" of the daily press and by the pamphlets of pseudo-scientists.

This brings me to the subject of telepathy, or the influence of mind on mind at a distance, a matter which contains considerable evidence among intelligent people. The hypothesis of telepathy was first broached as an explanation of the experience of many persons as to warning dreams, apparitions of absent friends and relatives who were dying, presentiments of visits, remarkable sympathies between people, apparently spontaneous expression of similar thoughts by individuals or peoples widely separated, and the like. If telepathy be true, our knowledge of physical conditions at the present day, such as modes of motion, interatomic ether, intermocular modulations, the sympathetic vibration of tuning forks and strings, etc., affords an easy scientific explanation of the phenomena. But have we evidence of the existence of telepathy sufficient to satisfy the most critical scientific mind, evidence as conclusive as that which leads us to accept as facts the common events of our every-day life, and the rarer facts accumulated by science in every field?

I have been at some pains to go over the evidence which has been gradually accumulated in relation to telepathy, and have even done some experimental work in this line. For instance, some years ago, Mr. Kennelly, then chief electrician of the Edison laboratory, and myself, made some experiments at that laboratory with a view to determining whether currents of varying vibration in the neighborhood of the head, might not be made by induction to set up molecular vibrations in the brain and thus rouse different thought processes into action. We each of us placed our head inside of huge coils of wire through which powerful alternating currents were passed with high frequency. The result was negative. Some further experiments upon the heads of lower animals made at the same time, however, demonstrated that the brain is so wonderfully insulated by the skull and scalp, which act so well as short curcuits, that probably the vibrations we employed upon ourselves did not reach our brains at all. Hence our negative results in these interesting experiments did not in any way invalidate the telepathic hypothesis. Now then, as to the evidence afforded by hallucinatory presentiments in dreams, these I take it are too vague and shadowy to appeal to any but superstitious and emotional people.

I think the best minds in the Society for Psychical Research do not attach much if any importance to dream-warnings. Dreams are so indefinite, so fleeting, so difficult to remember accurately, even if written down at once upon awaking, which is seldom the case; and if not immediately fixed upon paper, dreams are so plastic that they become extraordinarily modified by unconscious sophistication and dramatisation in direct ratio to the lapse of time between the event and its recital. Hallucinatory presentiments in the waking state or supposedly in the waking state are believed to have more value in this connection. But I have read many well authenticated examples of this kind, and it seems to me they cannot run the gauntlet of unbiased critical judgment. Incidents of this sort related to me by friends I regretfully reject, since the most conscientious people are unwittingly subject to the sources of error I have mentioned, illusory memories and the uncontrollable tendency to dramatic recreation of long-past events. But accounts written at or near the occurrence and corroborated by a creditable witness are more deserving of examination. These, however, are very rare, so rare indeed that chance coincidence may well serve to clear up their apparent mystery. It is possible that these so-called hallucinatory presentiments in the waking state are really subconscious, the mind lapsing momentarily to a lower plane of consciousness, just as dreams are believed to occur, not in deep sleep, but on the borderlands between sleep and waking. It would not be unnatural to suppose that during such a moment of obscured consciousness, the fact or voice of a dear relative or friend, so closely woven into all our subliminal associations, might rise like reality into that twilight of the mind. This indeed might happen not infrequently and all the more surely if there lurked any vague easily suggestible uneasiness in those subconscious levels, because of the illness of the absent one. Many inconsequential mirages of this nature pass unnoticed but immediately upon the occurrence of a catastrophe the presentiment is recalled, definitely linked with the event by an unconscious perversion of the sequences and facts, and welded into a stronger and stronger catenation with the lapse of time. I believe therefore that we have as yet no satisfactory evidence of the existence of telepathy, although I should be the last to deny the possibility of this or even greater marvels. We should keep our minds hospitably open to receive and welcome any strange guest bearing the proper credentials.

The word clairvoyance has a respectable significance, clear-seeing, intuitive sagacity—but it has also a savor of humbug and trickery. The astronomer is a true clairvoyant who foresees transits, eclipses and the paths of comets. The signal officer is a clairvoyant as regards storms and good weather. They see clearly what will happen through the sagacious eye of science. But the clairvoyance of the transmedium and the crystal visionary is a mixture of fraud, suggestion, autosuggestion and coincidence.

Spiritualism, or as my friend Prof. Hyslop, of Columbia, now terms it, spiritism, is not only a distinctively American invention, but Western New York may claim what honor is to be associated with its origin, for Rochester produced it in 1848. The tenets of this cult spread very rapidly, and curiously enough, Spain and the United States stand foremost among the nations where it has found most proselytes—a singular bond of sympathy. Spain publishes forty, and the United States about thirty periodicals dealing with spiritualistic philosophy and phenomena. These two countries together publish seven-tenths of the spiritualistic periodicals of the whole world. Why should two countries so radically different in point of age, freedom of opinion and diffusion of culture, be united by such a bond? Superstition and ignorance will immediately be named as the chief factors in Spain—why not also here? A man may have common sense and a considerable store of philosophy without knowing how to read and write. The converse of this is also true. He may read and write very well and be minus sense and philosophy. We may point proudly to our statistics of education as judged by the general knowledge of the three R's, but I fear that our plane of sense and philosophy coincides very closely with that of Spain. There may be other factors at work to keep us at this present level, but they are obscure. How else are we able to explain the enormous prevalence of spiritualism, theosophy, christian science, faith cure, factories of patent medicines and medical quackeries of all kinds? But I must not longer take up your time with these interesting phases of practical psychology. Few, if any of you present, would ask for an analysis of the evidence in relation to these subjects, having already determined by yourselves its triviality and inconclusiveness.

If I have made clear the seven sources of fallacy in evidence related to the material things with which our profession has to deal, it is readily seen that the sources of error in evidence connected with the immaterial world must be almost seventy times seven. But we shall continue the process of sifting and sifting evidence through the centuries and ever increasing the great pile of frauds laid bare, lies discovered, illusions corrected and delusions rejected, and at last when the pile is heaped high, what a bonfire of newspapers, pamphlets, books and apparatus shall be lighted by our descendents to celebrate their escape from the bondage of ignorance and superstition!

4 West Fiftieth Street.

